# Annual Hazard Rate of Recurrence in Middle Eastern Papillary Thyroid Cancer over a Long-Term Follow-Up

**DOI:** 10.3390/cancers12123624

**Published:** 2020-12-03

**Authors:** Abdul K. Siraj, Sandeep Kumar Parvathareddy, Zeeshan Qadri, Khawar Siddiqui, Saif S. Al-Sobhi, Fouad Al-Dayel, Khawla S. Al-Kuraya

**Affiliations:** 1 Human Cancer Genomic Research, Research Center, King Faisal Specialist Hospital and Research Center, P.O. Box 3354, Riyadh 11211, Saudi Arabia; asiraj@kfshrc.edu.sa (A.K.S.); psandeepkumar@kfshrc.edu.sa (S.K.P.); sqadri96@kfshrc.edu.sa (Z.Q.); 2 Department of Pediatric Hematology-Oncology, King Faisal Specialist Hospital and Research Center, P.O. Box 3354, Riyadh 11211, Saudi Arabia; ksiddiqui@kfshrc.edu.sa; 3 Department of Surgery, King Faisal Specialist Hospital and Research Center, P.O. Box 3354, Riyadh 11211, Saudi Arabia; sobhi@kfshrc.edu.sa; 4 Department of Pathology, King Faisal Specialist Hospital and Research Centre, P.O. Box 3354, Riyadh 11211, Saudi Arabia; dayelf@kfshrc.edu.sa

**Keywords:** annual hazard, recurrence, time-varying, papillary thyroid cancer, radioactive iodine

## Abstract

**Simple Summary:**

Tumor recurrence is a relatively common event in papillary thyroid cancer (PTC). The aim of this study was to analyze the time-varying pattern of recurrence in PTC using annual hazard function and establish a predictive model for early and late recurrences. We found a double-peaked pattern of annual hazard of recurrence, with the risk being highest during the first 5 years after surgery. We also found distinct clinico-pathological parameters that could independently predict the occurrence of early recurrence (first 5 years) as compared to late recurrence (after 5 years). Overall, this study highlights the importance of long-term follow-up of PTC patients. Furthermore, the findings of our study could help in establishing individualized treatment and surveillance plans in PTC patients.

**Abstract:**

Predicting the pattern of recurrence in papillary thyroid cancer (PTC) is necessary to establish optimal surveillance and treatment strategies. We analyzed changes in hazard rate (HR) for tumor recurrence over time in 1201 unselected Middle Eastern PTC patients. The changes in risk were further analyzed according to clinical variables predictive of early (≤5 years) and late (>5 years) recurrence using Cox regression analysis to identify patient populations that remain at risk. Tumor recurrence was noted in 18.4% (221/1201) patients. The annualized hazard of PTC recurrence was highest during the first 5 years (2.8%), peaking between 1 and 2 years (3.7%), with a second smaller peak between 13 and 14 years (3.2%). Patients receiving radioactive iodine (RAI) therapy had lower recurrence hazard compared to those who did not (1.5% vs. 2.7%, *p* = 0.0001). Importantly, this difference was significant even in intermediate-risk PTC patients (0.7% vs. 2.3%; *p* = 0.0001). Interestingly, patients aged ≥55 years and having lymph node metastasis were at persistent risk for late recurrence. In conclusion, we confirmed the validity of the double-peaked time-varying pattern for recurrence risk in Middle Eastern PTC patients and our findings could help in formulating individualized treatment and surveillance plans.

## 1. Introduction

Thyroid cancer is the most common endocrine cancer, and its incidence is increasing worldwide [1,2]. Nearly 20% of papillary thyroid cancer (PTC) patients who have no evidence of disease after initial treatment present with recurrence of disease during subsequent follow-up [3,4,5,6]. Although most recurrences in PTC are not fatal, they can be a great burden for patients, especially in a population with a high incidence rate for PTC, like Saudi Arabia [7].

The timing of recurrence in PTC varies considerably, being influenced by classical prognostic factors and adjuvant radioactive iodine (RAI) treatment strategies [8,9,10,11]. The average time to recurrence has been reported in the literature as being anywhere from 6 months to decades later [12,13,14]. However, it is only recently that clinical studies have begun to lay emphasis on late relapse in PTCs, with very little being known regarding the pattern of recurrence occurring beyond 10 years of follow-up [15]. Furthermore, most of the studies analyzing long-term outcomes in PTC have often focused on mortality rather than recurrence [16,17]. However, survival in PTCs is usually excellent and hence less informative about the natural history of disease after long-term follow-up [18,19,20]. The American Thyroid Association (ATA), in 2015, announced new treatment guidelines for differentiated thyroid cancer based on risk of recurrence [21]. Utilization of the ATA risk stratification has allowed for a more individualized approach to patient treatment [22,23,24,25]. Thus, it is important to understand changes in the risk of recurrence over time and to assess how clinical factors affect these changes. A better description of recurrence patterns, leading to a greater understanding of time-specific risk, could result in a more tailored therapeutic approach.

The purpose of this study was to analyze the hazard of recurrence after surgery for PTC patients from this ethnicity, where the prevalence of PTC is high in comparison to other populations, and to clarify the changes in hazard rate for recurrence over time in PTC patients. We also estimated the hazard rate of recurrence in RAI ablation patients from the entire cohort and with reference to the ATA risk categories. Finally, the risk factors for early and late recurrence were explored, offering unique opportunities to better define patterns of PTC recurrence.

## 2. Results

A total of 1201 patients were included in the analysis. Clinico-pathological characteristics of the study population are summarized in Table 1. Tumor recurrence was noted in 18.4% (221/1201) patients. The majority of the patients had regional recurrence (115/221; 52.0%). Distant metastasis was seen in 29.9% (66/221) cases, involving the lungs (50 cases; 75.8%), bone (9 case; 13.7%), both the lungs and bone (5 cases; 7.5%), brain (1 cases; 1.5%) and both lung and liver (1 case; 1.5%). Local recurrence was identified in 18.1% (40/221) cases, with 70% (28/40) of them occurring in the operated thyroid bed and the remaining 30% (12/40) in the residual thyroid tissue. Moreover, 87.3% (193/221) of recurrences occurred within the first 10 years after initial treatment, with the majority occurring within the first five years (144 cases; 65.2%), whereas 12.7% (28/221) of cases occurred between 10 and 20 years after surgery.

### 2.1. Recurrence Hazard Analysis in the Entire Cohort

**Figure 1:**
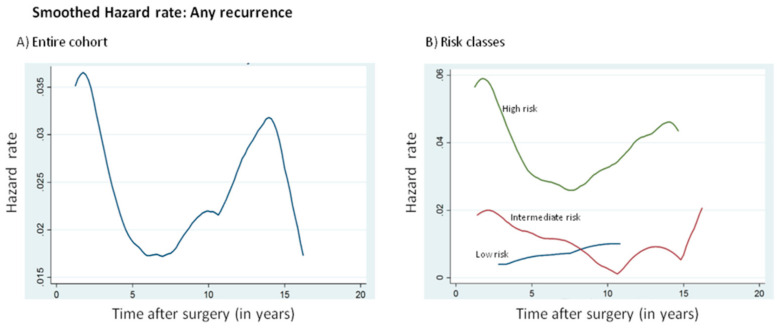
Annual hazard rate of cancer recurrence for the (**A**) entire population and (**B**) different risk classes.

The annual hazard curve of recurrence for the entire study population showed a double-peaked pattern, with peaks at 1–2 and 13–14 years after initial treatment (Figure 1A). The annualized hazard of PTC recurrence was highest during the first 5 years (2.8%), peaking at 2 years (3.7%). The hazard decreased to 1.8% between 5 and 10 years but increased again between 10 and 15 years (2.4%) (Table 2). When stratified based on ATA risk categories, the curves exhibited a double-peaked pattern in high- and intermediate-risk patients, but not in the low-risk patients, where it showed a single-peak pattern of recurrence. The recurrence peak emerged earlier for high- and intermediate-risk patients compared to the peak for low-risk patients, suggesting that low-risk PTC patients were less likely to recur early (Figure 1B).

### 2.2. Recurrence Hazard Analysis Based on RAI Status.

**Figure 2:**
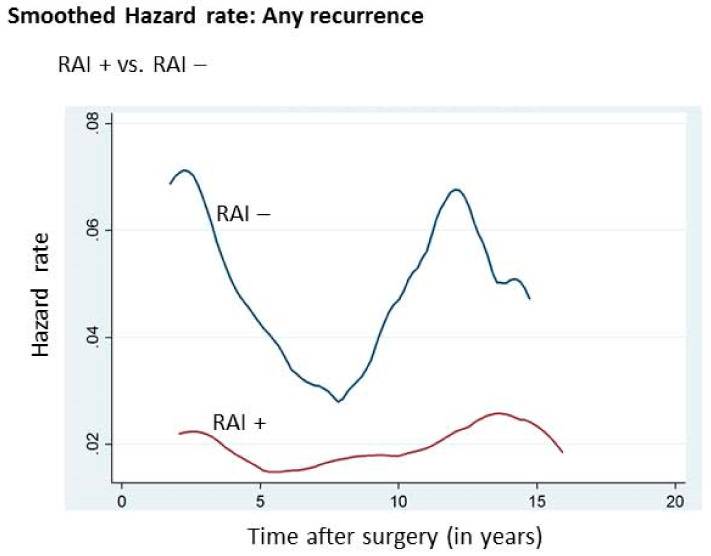
Annual hazard rate of cancer recurrence in the entire cohort according to RAI status.

Since the majority (78.0%; 937/1201, Table 1) of cases in our cohort received RAI therapy following initial surgery, we analyzed the annual hazard of recurrence based on the RAI therapy status. Throughout the follow-up period, patients receiving RAI therapy had a lower hazard of recurrence compared to those who did not receive RAI therapy (1.5% vs. 2.7%, *p* = 0.0001) (Table 3, Figure 2). On analyzing the annualized hazard of recurrence in 5-year intervals, difference was significantly lower in the RAI treated group compared to RAI untreated group between years 0 and 5 (2.2% vs. 5.7%; *p* = 0.0001) and 10 and 15 (2.0% vs. 6.0%; *p* = 0.0232), whereas it was not significant between years 5 and 10 (1.7% vs. 2.6%; *p* = 0.2544) and 15 and 20 (0.9% vs. 2.5%; *p* = 0.4307) (Table 4). A similarly significant difference in recurrence over time was noted for high- and intermediate-risk but not low-risk patients (Table 4).

**Figure 3:**
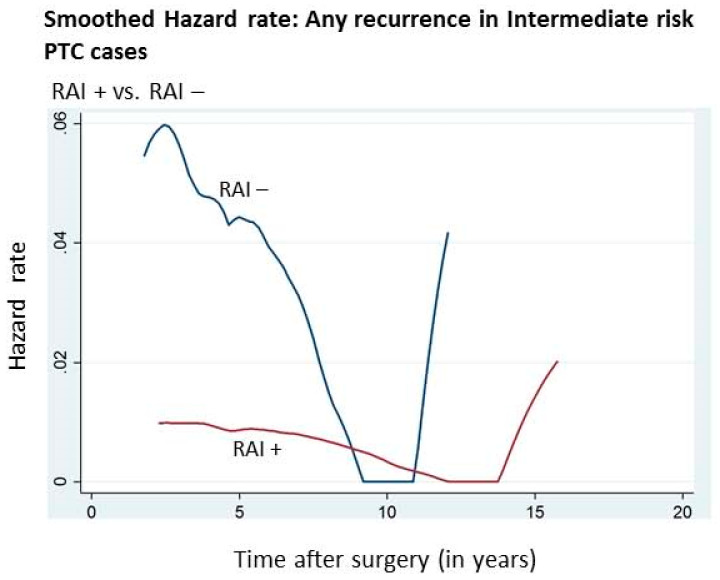
Annual hazard rate of cancer recurrence in the intermediate risk PTC according to RAI status.

Furthermore, since the role of RAI ablation in intermediate-risk PTC is controversial, we sought to analyze the annual hazard of recurrence with respect to RAI status in the intermediate-risk PTC. We found that patients with intermediate-risk PTC had a significantly higher hazard of recurrence in the RAI untreated group compared to the RAI treated group (2.3% vs. 0.7%; *p* = 0.0001) (Table 3, Figure 3).

### 2.3. Factors Predicting Early and Late Recurrence of PTC on Multivariate Analysis

Figure 1B shows a double-peaked pattern for high- and intermediate-risk patients, whereas Table 4 shows a significant difference in the recurrence rate over time for high- and intermediate-risk patients but not low-risk patients. Hence, we restricted the multivariate analysis using Cox regression to only high- and intermediate-risk patients. Since the first peak for both high- and intermediate-risk patients plateaued at around 5 years, we divided the recurrences into early (0–5 years) and late (>5 years) in order to analyze the factors that could predict these early and late recurrence. On Cox regression model analysis, male gender, age ≥ 55 years, T3-4 tumors and lymph node metastasis were predictors of early tumor recurrence in PTC (Table 5). Only age ≥ 55 years and lymph node metastasis were predictors of late tumor recurrence in PTC (Table 6).

## 3. Discussion

Risk analysis of tumor recurrence is highly important for the detection of recurrence, especially in PTC patients. In most of the existing studies, risk of recurrence has been analyzed by survival curves rather than using the hazard functions [26,27]. While survival curves only provide information on the cumulative time distribution of recurrence-free rate, hazard functions can depict the recurrence rate at any point in time among the remaining at-risk individuals [28].

In our study, the overall recurrence rate was relatively high. Although the majority (78%) of patients had received radioactive iodine (RAI), nearly one-fifth (18.4%) of patients suffered from disease recurrence in this study. This relatively high percentage is in concordance with other populations [13,29,30]. Interestingly, a recent study from a Japanese PTC cohort showed a similar recurrent rate despite only 1.5% of patients receiving RAI therapy [15]. The relatively high incidence of recurrence in this cohort could be due to the uniqueness of PTC in this population in that nearly 50% of patients presented with high-risk disease and only 15% had low-risk disease, which is not seen in most modern studies of western populations and could be attributed to genetics or differences in presentation and access to healthcare. Another reason for the high incidence of recurrence could be due to referral bias since patients with advanced disease are referred to our hospital from all over Saudi Arabia. Previous studies from Saudi Arabia have also reported a higher incidence of advanced disease [31,32], suggesting that thyroid cancer from Saudi Arabia could be more aggressive than in other parts of the world. 

The annual hazard curve of recurrence for the entire cohort showed a double-peaked pattern, with the first major surge reaching a peak during the second year after surgery, followed by another peak between 13 and 14 years. The annual hazard curves exhibited double-peaked distribution for high- and intermediate-risk patients and single-peaked distribution for low-risk patients. The recurrence peak for high- and intermediate-risk patients emerged earlier than low-risk patients, which suggests the importance of high surveillance and follow-up to detect early recurrence in this subset of patients. Furthermore, since low-risk PTC patients had a very low hazard of recurrence beyond 10 years of initial surgery, long-term follow-up may not be necessary for this group of patients. The double-peaked pattern of recurrence hazard in our study is in line with the tumor dormancy theory [33,34], which hypothesizes that micro-metastatic foci may exist in varying biological steady states, with most of them remaining dormant. However, this steady state may be disrupted by surgery, stimulating the switch from dormancy to growth, hence causing a sudden acceleration of metastatic process resulting in recurrence [35].

This double-peaked recurrence hazard pattern has been previously observed in several cancers [36,37,38,39]. Despite limited studies on the hazard of recurrence in thyroid cancer, a recent study by Dong et al. [15] in a cohort of 400 Japanese PTC patients showed triple-peaked annual hazard of recurrence with surges at 12, 22 and 29 years after initial surgery. Several factors might contribute to the difference in annual hazard curves between our study and Dong’s study. Sample size, follow-up timing, risk stratification, adjuvant RAI therapy given and ethnic differences might help in explaining these differences.

Little is known about the hazard of recurrence with respect to RAI status. Our data showed that patients receiving RAI therapy had a significantly reduced annual hazard of recurrence compared to those who did not receive RAI, particularly in high- and intermediate-risk patients (*p* = 0.0001). This highlights the clinical importance of giving RAI in intermediate-risk PTC patients. Giving RAI in intermediate-risk PTC has been a subject of controversy. On the one hand, studies have shown that RAI therapy could reduce recurrence and hence should be considered in intermediate-risk PTC patients [21,40], whereas others have suggested that RAI ablation may not have a beneficial role in decreasing the risk of recurrence in intermediate-risk PTC patients [41,42]. 

The present study is unique in that it explored the risk factors that could predict early and late recurrence using Cox regression analysis. This analysis revealed that PTC patients who were male, aged ≥55 years, with T3-4 tumors and lymph node metastasis were at high risk for early recurrence, whereas only age and lymph node metastasis were predictors of late recurrence. Our data suggest that a subset of patients who are male with T3-4 tumors might need more intensive surveillance in the initial 5 years following surgery, whereas those patients who are aged ≥55 years and have lymph node metastasis will have to be followed up for a longer period of time.

## 4. Materials and Methods

### 4.1. Patient Selection

One thousand four-hundred and sixty-six consecutive unselected PTC patients diagnosed between 1988 and 2015 at King Faisal Specialist Hospital and Research Centre (Riyadh, Saudi Arabia) were available to be included in the study. Cases were identified based on clinical history followed by fine needle aspiration cytology for confirmation. However, patients aged ≤18 years (*n* = 84), with a history of previous thyroidectomy (*n* = 144) or who were never free of disease (*n* = 37) were excluded from the study. After exclusion, 1201 patients were eligible and included in this study. The Institutional Review Board of the hospital approved this study and the Research Advisory Council (RAC) provided waiver of consent under project RAC # 2110 031. 

### 4.2. Clinico-Pathological Data

The baseline clinico-pathological data were collected from case records and have been summarized in Table 1. Extra-thyroidal extension was further classified as follows: ExT0, no extra-thyroidal extension; ExT1 (microscopic extra-thyroidal extension), microscopic invasion of tumor into perithyroidal soft tissues; ExT2 (gross extra-thyroidal extension), macroscopic invasion of tumor into perithyroidal soft tissues. Staging of PTC was performed using the eighth edition of American Joint Committee on Cancer (AJCC) staging system. Patients were stratified into low, intermediate and high risk based on 2015 ATA guidelines [21]. Thyroidectomies were divided into either total thyroidectomy or less than total thyroidectomy (subtotal, lobectomy). Overall, 78.0% (937/1201) of patients received radioactive iodine therapy following surgery. Low-risk patients received a mean cumulative RAI dosage of 110.2 mCi (SD = ±44.6 mCi), intermediate-risk patients received 135.6 ± 74.8 mCi and high-risk patients received 187.8 ± 136.4 mCi. Based on the ATA guidelines, tall cell, hobnail, columnar cell, diffuse sclerosing and insular variants were classified as aggressive variants, whereas classical and follicular variants were classified as non-aggressive variants.

### 4.3. Classification of Recurrence

Recurrence was defined as any newly detected tumor or metastatic lymph node based on ultrasound and/or imaging studies in patients who had been previously free of disease following initial treatment. Recurrence was classified according to the site, as follows: “local recurrence” if only the residual thyroid gland tissue or thyroid bed was involved; “regional recurrence” if central or lateral neck lymph nodes were involved; “distant recurrence” if disease was seen in soft tissues or lymph nodes at distant sites and visceral metastasis in other organs such as the lungs, liver, bones and brain. Biochemical recurrences were not considered for this study.

### 4.4. Follow-Up and Study Endpoints

Following initial surgery, low-risk PTC patients were followed up annually, intermediate-risk patients were followed up at 6-month intervals and high-risk patients were followed up at 3-month intervals. At each follow-up, neck ultrasound, thyroid function tests, thyroglobulin levels and thyroglobulin antibodies were performed. In addition, for high-risk patients, radioiodine scan and PET CT scan were performed at each follow-up to identify tumor recurrence. A biopsy confirmation (fine needle aspiration or histopathology) of tumor recurrence was obtained in 51.1% (113/221) cases. The remaining 48.9% (108/221) of cases were diagnosed by imaging studies alone. The median follow-up was 9.5 years (range 0.02–30.01 years). The primary study endpoint for our analysis was recurrence-free survival (RFS). RFS was defined as the time (in years) from date of initial surgery to the occurrence of any tumor recurrence (local, regional or distant). In the case of no recurrence, date of last follow-up was the study endpoint.

### 4.5. DNA Isolation and Sanger Sequencing Analysis

DNA samples were extracted from formalin-fixed and paraffin-embedded (FFPE) PTC tumor tissues utilizing Gentra DNA Isolation Kit (Gentra, Minneapolis, MN, USA) according to the manufacturer’s protocols, as elaborated in previous studies [43].

Sequencing of entire coding and splicing regions of exon 15 in BRAF gene, exon 2 and 3 in HRAS and NRAS genes among 1201 PTC samples was carried out using Sanger sequencing technology. Primer 3 online software was utilized to design the primers (available upon request, Primer3web v4.1.0, https://primer3.ut.ee/). PCR and Sanger sequencing analysis were carried out as described previously [44]. Reference sequences were downloaded from the NCBI GenBank and sequencing results were compared with the reference sequences by Mutation Surveyor V4.04 (Soft Genetics, LLC, State College, PA, USA).

### 4.6. Statistical Analysis

Annual hazard rates were estimated using the maximum likelihood estimate from piece-wise exponential model and Kernel smoothing method was used for graphical representation. To determine the independent prognostic factors for early and late recurrence, Cox proportional hazards regression model was used. Covariates for Cox regression analysis were selected if they were statistically significant on univariate analysis. All the variables were significant on univariate analysis, except for multifocality and histology. However, these two variables were included since they are integral to the ATA risk stratification of PTCs. Two-sided tests were used for statistical analyses, with a limit of significance defined as *p* value < 0.05. Statistical analyses were performed using Stata v9.0 (StataCorp Ltd., College Station, TX, USA) and SPSS v20.0 (SPSS, Chicago, IL, USA). 

## 5. Conclusions

This study confirms the validity of double-peaked time-varying pattern for risk of recurrence in Middle Eastern PTC patients. We therefore provide further support to the tumor dormancy hypothesis reported by Demicheli et al. [34]. Furthermore, according to the time distribution of recurrence hazard, we could formulate individualized treatment and surveillance plans in PTC patients.

## Figures and Tables

**Table 1:** Clinico-pathological variables for the patient cohort (*n* = 1201).

Clionico-Pathological Variables	*n* (%)
**Age at surgery (years)**	
<55	979 (81.5)
≥55	222 (18.5)
**Gender**	
Male	289 (24.1)
Female	912 (75.9)
**Histologic subtype**	
Classical variant	815 (67.9)
Follicular variant	248 (20.6)
Tall cell variant	114 (9.5)
Other variants	24 (2.0)
**Histologic subtype**	
Aggressive variants	138 (11.5)
Non-aggressive variants	1063 (88.5)
**Tumor size (cm)**	
≤4	601 (50.8)
>4	582 (49.2)
**Multifocality**	
Yes	599 (49.9)
No	602 (50.1)
**Extrathyroidal extension**	
ExT0	700 (58.3)
ExT1	411 (34.2)
ExT2	90 (7.5)
**Number of lymph nodes involved**	
≤5	913 (80.3)
>5	224 (19.7)
**Lymph node metastasis**	
N0	565 (47.1)
N1a	165 (13.7)
N1b	407 (33.9)
Nx	64 (5.3)
**Extent of thyroidectomy**	
Total thyroidectomy	1030 (85.8)
Less than total thyroidectomy	171 (14.2)
**Risk class**	
Low	183 (15.2)
Intermediate	430 (35.8)
High	588 (49.0)
**Recurrence**	
Yes	221 (18.4)
Local	40 (3.3)
Regional	115 (9.6)
Distant	66 (5.5)
No	980 (81.6)
**TNM stage**	
I	957 (79.7)
II	160 (13.3)
III	63 (5.2)
IV	21 (1.8)
**Radioactive iodine therapy**	
Yes	937 (78.0)
No	264 (22.0)
**Status at last follow-up**	
Alive	1162 (96.8)
Deceased	39 (3.2)
Due to Papillary thyroid cancer	25 (2.1)
Due to other reasons	14 (1.1)
**BRAF mutation**	
Present	675 (56.2)
Absent	470 (39.1)
Unknown	56 (4.7)
**NRAS mutation**	
Present	77 (6.4)
Absent	1063 (88.5)
Unknown	61 (5.1)
**HRAS mutation**	
Present	22 (1.8)
Absent	1115 (92.9)
Unknown	64 (5.3)
**Follow-up duration (years)**	
Median	9.50
Range	0.01–30.01

Clinico-pathologial variables are highlighted in bold.

**Table 2:** Annual hazard of recurrence estimated within 5-year intervals in the entire cohort and different risk classes.

Outcome	Hazard (%; SE)				*p* Value
	Years 0–5	Years 5–10	Years 10–15	Years 15–20	
RFS					
All patients	2.8 (0.2)	1.8 (0.3)	2.4 (0.5)	1.1 (0.5)	0.021 *
Low Risk	0.2 (0.2)	1.0 (0.4)	0.5 (0.5)	0.0 (0.0)	0.354
Intermediate Risk	1.8 (0.3)	0.9 (0.3)	0.4 (0.4)	1.4 (1.4)	0.177
High Risk	4.6 (0.4)	2.8 (0.5)	3.9 (0.8)	1.4 (0.8)	0.003 *

RFS—Recurrence-free survival; *, significant *p* value.

**Table 3:** Annual hazard of recurrence in the entire cohort and in different risk categories with respect to radioactive iodine (RAI) ablation status.

Groups	RAI Status	Annual Hazard (%; SE)	*p* Value
Entire cohort	RAI given	1.5 (0.1)	0.0001 *
	RAI not given	2.7 (0.3)	
High risk	RAI given	2.9 (0.3)	0.0001 *
	RAI not given	5.8 (0.8)	
Intermediate risk	RAI given	0.7 (0.1)	0.0001 *
	RAI not given	2.3 (0.5)	
Low risk	RAI given	0.4 (0.2)	0.8427
	RAI not given	0.5 (0.3)	

*, significant *p* value.

**Table 4:** Annual hazard of recurrence estimated within 5-year intervals based on RAI ablation status in the overall cohort and different risk categories.

Outcome	Hazard (%; SE)			
	Years 0–5	Years 5–10	Years 10–15	Years 15–20
RFS (Overall cohort)				
RAI +	2.2 (0.2)	1.7 (0.3)	2.0 (0.5)	0.9 (0.5)
RAI −	5.7 (0.8)	2.6 (0.8)	6.0 (2.2)	2.5 (2.5)
*p* value	0.0001 *	0.2544	0.0232 *	0.4307
RFS (High risk)				
RAI +	3.6 (0.4)	2.5 (0.5)	3.2 (0.8)	0.7 (0.7)
RAI −	10.5 (1.7)	6.0 (2.4)	20.0 (7.8)	40.0 (0.0)
*p* value	0.0001 *	0.0229 *	0.0001 *	NA
RFS (Intermediate risk)				
RAI +	1.1 (0.3)	0.7 (0.3)	0.0 (0.0)	1.7 (1.7)
RAI −	4.8 (1.2)	2.2 (1.3)	3.3 (3.3)	0.0 (0.0)
*p* value	0.0004 *	0.0840	0.0003 *	0.6301
RFS (Low risk)				
RAI +	0.2 (0.2)	1.1 (0.6)	0.0 (0.0)	0.0 (0.0)
RAI −	0.4 (0.4)	0.7 (.7)	1.6 (1.6)	0.0 (0.0)
*p* value	0.6693	0.6724	0.1708	0.6693

RFS—Recurrence-free survival. *, significant *p* value.

**Table 5:** Cox regression model analysis for prediction of early tumor recurrence (0–5 years).

Variables	Characteristic	Estimate (SE)	Hazard Ratio	95% CI	*p* Value
Gender	Male (vs. Female)	−0.424 (0.190)	0.654	0.451–0.949	0.025 *
Age	≥55 years (vs. <55 years)	0.798 (0.212)	2.222	1.465–3.369	<0.001 *
Tumor size	T3-4 (vs. T1-2)	0.796 (0.264)	2.216	1.320–3.720	0.003 *
Lymph node metastasis	N1 (vs. N0)	0.875 (0.218)	2.399	1.565–3.676	<0.001 *
Multifocality	Yes (vs. No)	−0.148 (0.186)	0.863	0.599–1.242	0.296
Extra-thyroidal extension	Microscopic (vs. absent)	0.276 (0.261)	1.317	0.791–2.195	0.290
	Gross (vs. absent)	0.522 (0.408)	1.685	0.758–3.764	0.201
Histology	Aggressive (vs. non-aggressive)	−0.192 (0.272)	0.825	0.484–1.406	0.480
Stage	III–IV (vs. I–II)	0.389 (0.331)	1.476	0.772–2.822	0.239
BRAF mutation	Present (vs. absent)	−0.169 (0.206)	0.845	0.564–1.264	0.412
Radioactive iodine	Given (vs. not given)	−1.341 (0.197)	0.262	0.178–0.385	<0.001 *

* Significant *p* value.

**Table 6:** Cox regression model analysis for prediction of late tumor recurrence (>5 years).

Variables	Characteristic	Estimate (SE)	Hazard Ratio	95% CI	*p* Value
Gender	Male (vs. Female)	−0.491 (0.275)	0.612	0.357–1.049	0.074
Age	≥55 years (vs. <55 years)	1.468 (0.291)	4.339	2.454–7.672	<0.001 *
Tumor size	T3-4 (vs. T1-2)	0.783 (0.419)	2.188	0.962–4.979	0.062
Lymph node metastasis	N1 (vs. N0)	0.862 (0.302)	2.368	1.311–4.277	0.004*
Multifocality	Yes (vs. No)	−0.264 (0.262)	0.768	0.459–1.284	0.314
Extra-thyroidal extension	Microscopic (vs. absent)	0.081 (0.400)	1.084	0.495–2.375	0.841
	Gross (vs. absent)	−0.294 (0.827)	0.746	0.147–3.773	0.723
Histology	Aggressive (vs. non-aggressive)	−0.114 (0.456)	0.893	0.365–2.183	0.803
Stage	III–IV (vs. I–II)	1.232 (0.700)	3.430	0.870–13.512	0.078
BRAF mutation	Present (vs. absent)	0.561 (0.333)	1.753	0.912–3.369	0.092
Radioactive iodine	Given (vs. not given)	−1.391 (0.328)	0.249	0.131–0.473	<0.001 *

* Significant *p* value.
